# A Ministry of Health and Its Operations

**Published:** 1917-11-10

**Authors:** 


### HEALTH PROBLEMS AND THE STATE.

### A Ministry of Health and its Operations.

Just now the public mind is mainly occupied with the most effective method of overcoming the enemies that are without. But the events of the day have also spread more widely a knowledge of the enemies that are within, and a fairly widespread resolve is framing itself that these, too, if civilisation is to justify itself, must be overcome. Perhaps most of the thinking as to ways and means is being prosecuted by those who rule over us. But our pastors and masters may be sure that there is a firm public mind set upon improvements in various directions, and there will be no lack of pressure when the appropriate time comes. The important thing is to develop guidance for this deep-seated conviction, and our legislative prescribers may be reminded that the first duty of a physician is to be careful to do no harm. They will certainly be expected to do something. May the fates forefend that they do no mischief! A writer with a large experience of men and affairs has recently told us that "it should be the object of all reasonable people to be governed as little as possible." But the wind just now certainly does not set in this direction, and there is a mean to be attempted which may well fail to please either of the extreme parties.

A recent discussion at the Royal Institute of Public Health gives some indication of proposals which are being cultivated, and it derives some importance from the speakers who took part in it. The Minister of Education, who presided, gave a dramatic account of the legislative fate of the British child who comes into the world under the ægis of the Central Midwives Board and the Lords of the Privy Council. A month or two later he suffers the care of a voluntary association, which may, or may not, be subsidised by the Board of Education or the Local Government Board. Next, at the school age, he is inspected by the School Medical Service under the authority of the Board of Education, and he continues to enjoy this advantage up to fourteen years of age. Now arrives a brief interval, from fourteen to sixteen years, when he escapes any special public health control. But at sixteen years there arrives the Insurance Commission, with at various dates the possible intervention of the Factory Department of the Home Office, the Ministry of Munitions, the Local Pensions Committee, the Poor-Law Guardians, or the authorities charged with the care of the mentally defective. There will be general agreement with Mr. Fisher's conclusion that such a programme presents large administrative difficulties. Such a recital moved at least one of the speakers in the discussion to express the view that the people are in danger of being "welfared" to death, and that the working classes are being worried into insanity by the supervision of multiple and competing authorities. But even this speaker agreed that the solution was to be found in a Ministry of Health, though he appeared to think that the chief virtue of the proposal was its capacity for suppressing the lesser fry. The chairman himself, with appropriate impartiality, left the whole question an open one, but submitted that the practical issue was not whether the arrangements he described constitute an ideal scheme, but whether in practice and as a matter of fact they work economically and without friction.

To these questions Major Waldorf Astor, who opened the discussion, gave an unhesitating reply. As is well known, Major Astor has a large knowledge of public health problems. Before the war he presided over a semi-official committee which was composed partly of members of Parliament and partly of practical medical administrators, and which conducted a careful inquiry into the whole subject. His account of the work of this committee is of great interest. At first opinion decidedly favoured the organisation of a new Ministry of Health, to be concerned with all public health activities. But later it was judged easier, quicker, and probably more effective to build on an existing Department. Reviewing the possibilities in this direction the committee decided that there were only two effective competitors—the Insurance Commission and the Local Government Board. At first the former seemed to be the more attractive, but again second thoughts carried it and the choice was made of the Local Government Board. It is true that this Board is loaded with many non-health functions relating, for example, to unemployment, roads, loans, and the rest. Still it includes a definite health organisation, and the committee's proposal was that this should be developed. A more important criticism arises from the association of the Local Government Board with the administration of the Poor Law, for all will agree that any public health service must escape the mark of pauperism. It is hoped that a change of name may help to banish the risk of prejudice, and the new title suggested is the Ministry of Public Welfare and Local Government. This is for the starting-point. Future developments may perhaps get a complete separation between health and non-health functions, but in the meantime it is necessary to utilise existing machinery and to secure by this the gradual inclusion of all public health activities under one and the same Ministry.

Now this proposition is clearly one which has been well thought out, and in view of the sanction it enjoys it cannot be lightly dismissed. At the same time, as the later discussion on Major Astor's paper shows, while there is general agreement that a Ministry of Health is a necessity, not everyone will accept the proposal to raise the Local Government Board to this position. Strong claims are advanced for an entirely new organisation, which will take over the health functions of all the Departments, including those of the Local Government Board, and this has been the view which has been most strongly pressed in medical circles. Still again, there is the plan advocated by Dr.

**HEALTH PROBLEMS AND THE STATE**—(*cont.*).

Brend, according to which the new Ministry of Health would be a more or less detached critic of the various public health performances of the other Government Departments, and would have for its own main motive the culture of statistical and precise information relative to the occurrence of disease and to the conditions which promote health in the community. The medical profession have, of course, the highest interest in this question, and some organisation of medical opinion in the matter is of acute importance. In some quarters it is urged that the existing pressure on the profession and the absence of so many members on military service make the whole discussion inopportune and compel the postponement of any practical steps. We doubt if this argument will receive much attention, though certainly it has a very plausible quality. So far as possible we have to be prepared for peace, and this means movement while yet the war continues. The Ministry of Health Bill may suffer delay for political reasons, but it will hardly halt because the doctors cannot find time to give it their attention. Wise men, even against their better judgment, are often compelled to legislate in haste because they were not wise enough, or not free enough, to legislate at leisure. On the functions and powers of the new Ministry, whatever be its point of departure, the medical profession will certainly have much to say. Medical officers of health may quite well encourage a development of control and interference which private practitioners would regard as intolerable. The former are part of the official machine, and they see clearly what benefits have followed the organisation and co-ordination of their work. It is probably not easy for them to appreciate the very different conditions which attend the work of the family practitioner. The latter stands, not on an official basis but on a personal one, and his immediate care—and rightly so—is the sick and suffering individual. Hence an amount of Government interference which might be helpful in preventive medicine would be intolerable if applied to private practice. The Insurance Act has brought the majority of private practitioners under the stress of one bureaucracy, and there is little enthusiasm for an extension of the discipline. There is good ground to believe that a vigorous effort will be made to institute a State Medical Service when the Ministry of Health Bill takes practical shape, and it is supposed that the doctors who have experienced the delights of official direction in the R.A.M.C. will favour the scheme. In any event it may be taken as certain that any such proposal will meet with strong opposition both from the profession and the public, and that while there may be active dissentients from each camp these will not be the only voices to be heard. Sufficient has been said to show that relative to the medical profession any proposed legislation dealing with the establishment of a Ministry of Health will demand the closest scrutiny. There are large public interests at stake, and these will be considered by the profession acting as citizens having special qualifications for judging such matters. In addition, immediate professional interests may be involved, and though the real welfare of the community is never opposed to these, not every ambitious reformer perceives this truth. We come back to our original point. Doctors and laymen must both recognise that something has to be done. This "something" if rightly directed may mean much benefit, but unless wisdom sits at the council "something" may be a source of endless mischief.

